# 5-Lipoxygenase-Activating Protein as a Modulator of Olanzapine-Induced Lipid Accumulation in Adipocyte

**DOI:** 10.1155/2013/864593

**Published:** 2013-05-25

**Authors:** Svetlana Dzitoyeva, Hu Chen, Hari Manev

**Affiliations:** Department of Psychiatry, Psychiatric Institute, University of Illinois at Chicago, 1601 West Taylor Street, MC912, Chicago, IL 60612, USA

## Abstract

Experiments were performed in 3T3-L1 preadipocytes differentiated *in vitro* into adipocytes. Cells were treated with olanzapine and a 5-lipoxygenase (5-LOX) activating protein (FLAP) inhibitor MK-886. Lipid content was measured using an Oil Red O assay; 5-LOX and FLAP mRNA content was measured using quantitative real-time PCR; the corresponding protein contents were measured using quantitative Western blot assay. Olanzapine did not affect the cell content of 5-LOX mRNA and protein; it decreased FLAP mRNA and protein content at day five but not 24 hours after olanzapine addition. In the absence of MK-886, low concentrations of olanzapine increased lipid content only slightly, whereas a 56% increase was induced by 50 **μ**M olanzapine. A 5-day cotreatment with 10 **μ**M MK-886 potentiated the lipid increasing action of low concentrations of olanzapine. In contrast, in the presence of 50 **μ**M olanzapine nanomolar and low micromolar concentrations of MK-886 reduced lipid content. These data suggest that FLAP system in adipocytes is affected by olanzapine and that it may modify how these cells respond to the second-generation antipsychotic drugs (SGADs). Clinical studies could evaluate whether the FLAP/5-LOX system could play a role in setting a variable individual susceptibility to the metabolic side effects of SGADs.

## 1. Introduction

Serious side effects hamper pharmacological treatment of psychiatric illnesses such as schizophrenia and bipolar disorder. All currently used second-generation antipsychotic drugs (SGADs) including olanzapine are capable of triggering significant weight gain associated with adverse metabolic alterations [1, 2]. It has been proposed that these side effects are caused by a combination of factors including increased fat deposition [3] and appetite stimulation [4]. Interestingly, it was noted that SGADs are capable of increasing adiposity even in the absence of significant body weight gain [5–7] presumably by drug-impaired lipolysis [7, 8] and/or a direct stimulatory action of SGADs on adipocytes [9–11]. In the therapy of psychiatric patients with SGADs, a better understanding of the mechanisms that lead to this clinical problem is needed to identify the risk factors that facilitate and exacerbate these side effects of SGADs and to develop methods and therapies to prevent and/or treat their occurrence.

 Recently, an *in vitro* model of 3T3-L1 cells has been developed to study the effects of olanzapine on peripheral adipogenesis [10]. Unrelated studies with this cell culture model have shown that the enzymatic pathway of 5-lipoxygenase (5-LOX), which typically is involved in the association between adipose tissues and inflammation [12, 13], may contribute to lipid dysfunction [14]. Interestingly, the olanzapine-induced enlargement of adipose tissue also has been associated with inflammation [15].

5-LOX gene polymorphism and the associated 5-LOX enzyme deficiency occur generally in humans and have been noted as an example of pharmacogenetics [16]. In mice, a 5-LOX deficiency (e.g., gene knockdown) is accompanied by increased adiposity and elevated plasma levels of leptin, a factor produced by adipocytes that acts in the brain [17, 18]. For its full activity, 5-LOX translocates to the nuclear membrane where it interacts with a transmembrane protein five lipoxygenase activating protein (FLAP) [19]. Both in human [20] and mouse [14] adipose tissue, FLAP expression is increased in obesity. Furthermore, a high-fat diet in mice caused a transient increase in the FLAP mRNA levels (after a 4-week treatment; returning to the basal levels at weeks 6 and 8) in aortic endothelium [21]. On the other hand, an acute fat load in healthy humans reduced FLAP expression in peripheral blood mononuclear cells [22]. Increased FLAP levels in adipose tissue are associated with increased levels of 5-LOX products, for example, leukotriene LTB_4_ that is capable of inducing the NF-*κ*B signaling pathway in adipocytes [14]. In obesity, a beneficial effect of pharmacological FLAP inhibition has been attributed to an induction of AMP-activated protein kinase phosphorylation and a concomitant decrease in hormone-sensitive lipase activity accompanied by reduced steatosis [14].

To our knowledge, the 5-LOX system has not been investigated in relation to the SGADs treatment. In this work, we used the 3T3-L1 cell culture model to investigate the putative association of the 5-LOX/FLAP system with olanzapine-triggered lipid accumulation. The preliminary results of this study have been reported in an abstract form [23].

## 2. Material and Methods

### 2.1. Cell Cultures and Drug Treatment

The 3T3-L1 preadipocytes (ATCC, American Type Culture Collection) were grown at 37°C, 5% CO_2_ air, in 35 × 10 mm tissue culture dishes (Falcon, Fisher) containing 2 mL of high glucose Dulbecco's modified Eagle's medium (DMEM, GIBCO) supplemented with 10% fetal bovine serum (FBS, Atlanta Biologicals). When the plated preadipocytes reached confluence, they were differentiated into adipocytes by changing the culture medium to DMEM/10% FBS medium supplemented with 1 *μ*g/mL insulin, 1 *μ*M dexamethasone, and 0.5 mM 3-isobutyl-1-methylxanthine (IBMX) (all from Sigma). This was considered day 0 *in vitro*. Cells were maintained *in vitro* for 5 days changing their medium at days 2 and 4 with fresh DMEM/10% FBS medium supplemented with 1 *μ*g/mL of insulin. Penicillin/streptomycin solution (1 : 100; GIBCO, Grand Island, NY, USA) was added with every medium addition as a contamination preventive measure. The experimental drugs olanzapine (Sequoia Research Products Ltd., Pangbourne, UK) and MK-886 (Cayman Chemical, Ann Arbor, MI, USA) or their vehicle (1 : 1000 dimethyl sulfoxide, DMSO; Sigma) were added at day 0 and in the refreshment medium at days 2 and 4 (adapted from [10]).

### 2.2. Oil Red O Assay

The culture medium was gently removed with a transfer pipette, and 1 mL of 12% formaldehyde in phosphate buffer saline was applied on top of the cells. After 20 min, cells were rinsed three times with 2 mL of distilled water and covered with 1 mL of Oil Red O (Sigma) solution (0.5% Oil Red O in isopropanol diluted with water 3 : 2 and filtered through a 0.45 *μ*m filter) for 30 min at room temperature. The Oil Red O solution was discarded with a transfer pipette, and cells were rinsed three times with 2 mL of distilled water. After removing all water, Oil Red O dye captured by intracellular lipids was recovered with 400 *μ*L of isopropanol applied drop by drop on top of the cells. The resulting solution was transferred into microcentrifuge tubes and its absorbance values were measured in a microplate reader (Bio-Rad, Model 550) in a single wavelength mode with a 520 nm filter. For the visualization of the Oil Red O, staining cells were grown on glass cover slips. They were fixed and stained as described above. Microphotographs were taken after transferring the cover slips onto a glass slide and covered them with a mounting solution.

### 2.3. RNA Extraction and Quantitative Real-Time PCR

Total RNA was extracted with TRIzol reagent (Invitrogen Carlsbad, CA, USA) according to the manufacturer's instructions and treated with DNase (Ambion Inc., Austin, TX, USA). Thereafter, 1 *μ*g of RNA was reverse transcribed with M-MLV reverse transcriptase (Invitrogen, Carlsbad, CA, USA). Random hexamer primers and dNTPs were purchased from Fermentas (Fermentas Inc., Glen Burnie, MD, USA). The quantitative real-time PCR was performed on Stratagene Mx3005P QPCR System (Agilent Technologies) with a PCR SYBR Green Master mix (Fermentas Inc., Glen Burnie, MD, USA). Primers used: 5-LOX forward 5′-attgccatccagctcaaccaaacc-3′, reverse 5′-tggcgataccaaacacctcagaca-3′; FLAP forward 5′-ccacaaggtggagcatgaaagcaa-3′, reverse 5′-aaacaggtacatcagtccggcgaa-3′; cyclophilin forward 5′-agcatacaggtcctggcatcttgt-3′, reverse 5′-aaacgctccatggcttccacaatg-3′. Data were normalized against the corresponding cyclophilin internal control and presented as a coefficient of variation calculated with a formula 2^−[Δ*Ct*(target)−Δ*C*(input)]^ [24].

### 2.4. Western Blot Assay

Cells were homogenized in 200 *μ*L ice cold RIPA buffer with protease inhibitors (5 *μ*g/mL aprotinin, 5 *μ*g/mL leupeptin, 5 *μ*g/mL pepstatin, 1 mM phenylmethanesulfonylfluoride) and then centrifuged at 10000 g for 15 min. The supernatant was stored at −80°C. After measuring protein concentration with a BCA protein assay kit (Pierce, Rockford, IL, USA), samples of 30 *μ*g proteins were boiled in loading buffer and run on 7.5% or 10% gels. Proteins were then transferred to nitrocellulose membranes. After blocking by 5% nonfat dry milk for 1 h, the membranes were incubated overnight at 4°C with rabbit anti-FLAP antibody (1 : 500, Santa Cruz, Santa Cruz, CA, USA), mouse anti-5-LOX antibody (1 : 1000, BD Bioscience, San Jose, CA, USA), or mouse anti-*β*-actin antibody (1 : 5000, Sigma, St. Louis, MO, USA). Thereafter, blots were incubated for 2 h with horseradish-peroxidase-linked secondary antibodies. The membranes were developed with an ECL Kit (Amersham). Band densities were quantified using NIH ImageJ software. The optical density of FLAP and 5-LOX bands was corrected by the corresponding *β*-actin bands.

### 2.5. Statistics

Statistical analyses were performed using SPSS software (SPSS Inc., Chicago, IL, USA). Data (shown as mean ± SEM) were analyzed by one-way analysis of variance (ANOVA) followed by post hoc multiple comparison with Bonferroni correction or Student's *t*-test. Significance was accepted at *P* < 0.05.

## 3. Results

**Figure 1:**
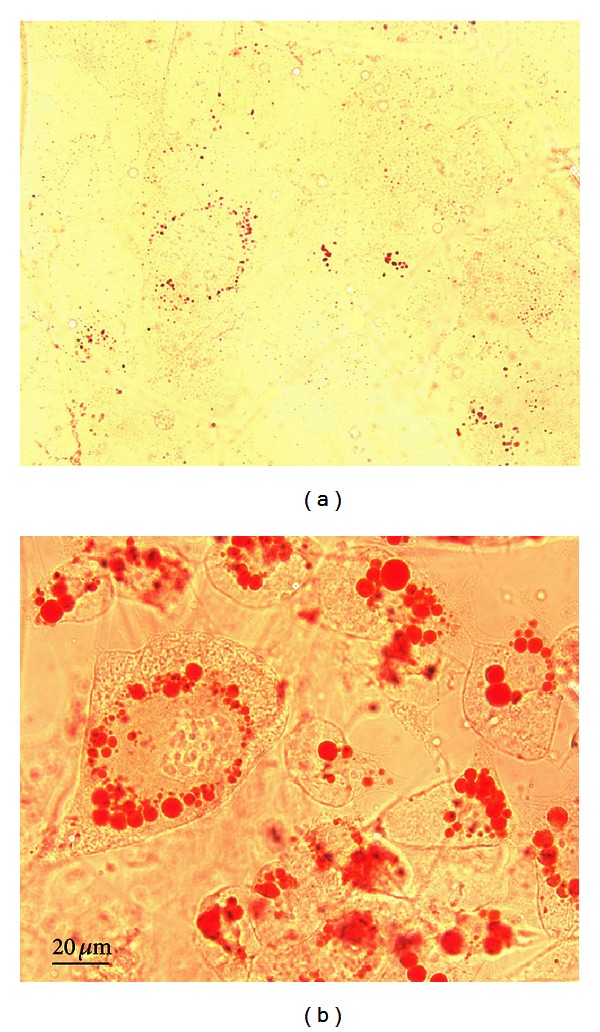
Oil Red O staining in undifferentiated 3T3-L1 cells (a) and after 5 days *in vitro* of adipocyte differentiation (b). Note the red/dark staining of oil droplets in B (×40 objective lens).

**Figure 2:**
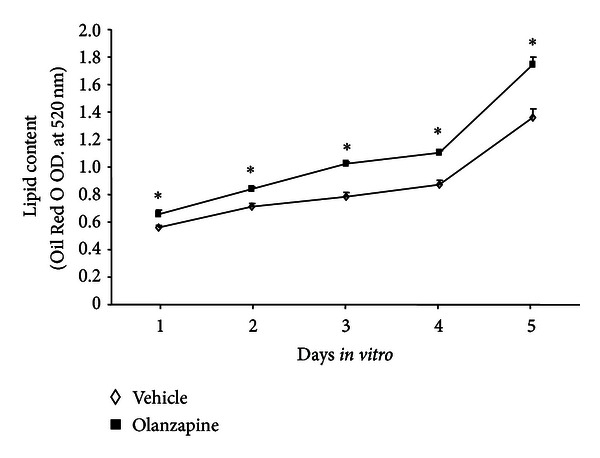
Effect of olanzapine on intracellular lipid content measured by a quantitative Oil Red O assay. 3T3-L1 cells were differentiated into adipocytes in the presence and absence of 50 *μ*M olanzapine. Samples (3 culture dishes per treatment and time point) were collected daily starting 24 hours after the initiation of the differentiation protocol. Results (mean ± SEM) are expressed as optical density (O.D.) readings at 520 nm. At each time point, the values of olanzapine-treated cultures differed significantly from the values measured in corresponding vehicle-treated cultures (*P < 0.05; *t*-test).

**Figure 3:**
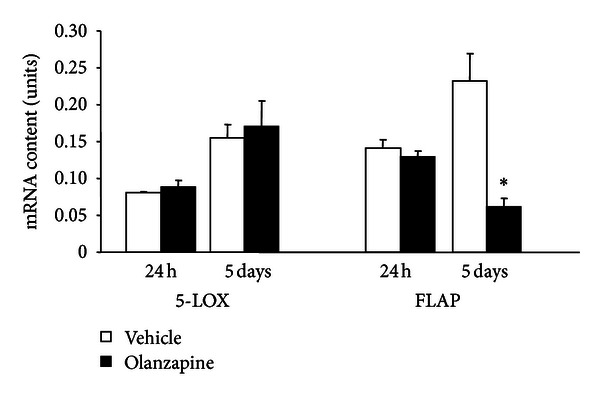
Effect of olanzapine treatment on 5-LOX and FLAP mRNA contents. 3T3-L1 cells were differentiated into adipocytes in the presence and absence of 50 *μ*M olanzapine. Samples (3 culture dishes per treatment and time point) were collected 24 hours and 5 days after the initiation of the differentiation protocol. The content of 5-LOX and FLAP mRNAs was quantified with a real-time PCR. Data (mean ± SEM) are expressed as units (coefficient of variation normalized to the corresponding cyclophilin internal control); *P < 0.05 versus the corresponding vehicle-treated control.

**Figure 4:**
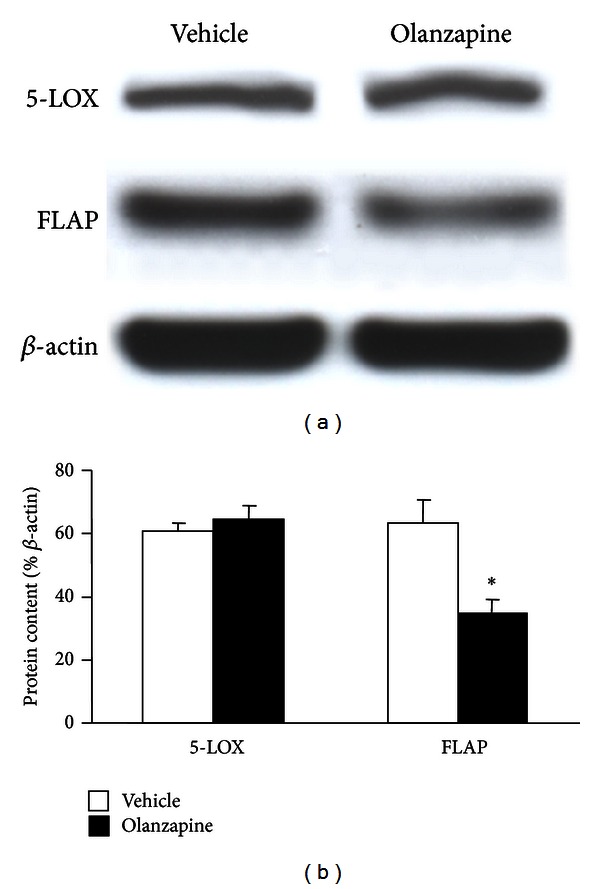
Effect of olanzapine treatment on 5-LOX and FLAP protein contents. 3T3-L1 cells were differentiated into adipocytes in the presence and absence of 50 *μ*M olanzapine. Samples were collected 5 days after the initiation of the differentiation protocol. The content of 5-LOX and FLAP proteins was assayed by quantitative Western blot. The optical density of the 5-LOX and FLAP bands was corrected by the density of the corresponding *β*-actin bands (examples shown in a). The results (b) are expressed as a percentage of the corresponding *β*-actin values (mean ± SEM; *n* = 5); *P < 0.05 versus the corresponding vehicle-treated control.

**Figure 5:**
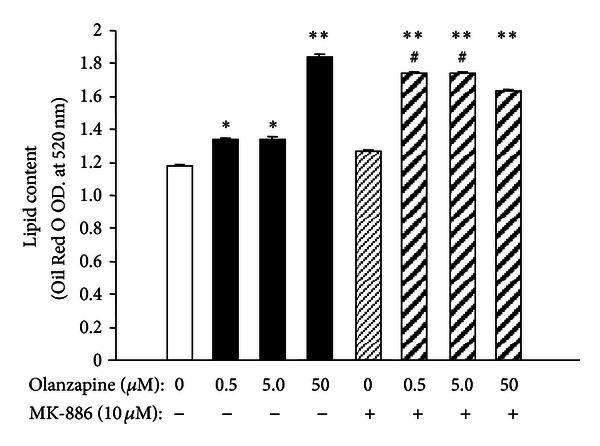
Effect of 10 *μ*M MK-886 in the presence of increasing concentrations of olanzapine on intracellular lipid content. 3T3-L1 cells were differentiated into adipocytes in the presence and absence of indicated drugs. Samples were collected 5 days after the initiation of the differentiation protocol. Results (mean ± SEM; *n* = 4) are expressed as optical density (O.D.) readings at 520 nm. *P < 0.05, **P < 0.001 versus the corresponding group without olanzapine; ^#^ *P* < 0.001 versus the corresponding group without MK-886.

To study the *in vitro* effects of olanzapine, we used a previously published protocol [10] for olanzapine-increased adipogenesis in 3T3-L1 cells. With this protocol, the preadipocytes, which expressed only a weak lipid staining (Figure 1(a)), in 5 days, differentiate into oil droplet-enriched adipocytes (Figure 1(b)). Addition of olanzapine to the culture medium of cells undergoing adipocyte differentiation resulted in an increase of lipid content in these cells (Figure 2). The difference in lipid content between olanzapine-treated and vehicle-treated cultures was significant 24 hours after olanzapine addition and remained so at each day of *in vitro* treatment, that is, until day five (Figure 2). In this model, olanzapine treatment did not affect the cell content of 5-LOX mRNA; however, it decreased FLAP mRNA content at day five but not 24 hours after olanzapine addition to the culture medium (Figure 3). The inhibitory effect of olanzapine on FLAP expression was confirmed by quantitative Western blot assay. Hence, the content of FLAP protein at day five of olanzapine treatment was significantly lower compared to vehicle-treated controls. The content of 5-LOX protein was not affected by olanzapine (Figure 4). Addition of a high concentration of FLAP inhibitor MK-886 (10 *μ*M) during the 5 days of olanzapine treatment enhanced the adipogenic effect of olanzapine (Figure 5). Hence, in the absence of MK-886, low concentrations of olanzapine (0.5 and 5 *μ*M) increased lipid content only by about 13% (compared to about a 56% increase induced by 50 *μ*M olanzapine), whereas in the presence of 10 *μ*M MK-886, these concentrations of olanzapine produced lipid increases comparable to the increase caused by 50 *μ*M olanzapine (Figure 5). In contrast, low concentrations of MK-886 (0.1–3 *μ*M) reduced the lipid content of cells treated with the high 50 *μ*M concentration of olanzapine (Table 1).

## 4. Discussion

In this study, we show evidence that exposure of differentiating preadipocytes to olanzapine, in addition to increasing their accumulation of lipids, alters the expression of FLAP and that a FLAP inhibitor, MK-886, used in nanomolar and low micromolar concentrations decreases the adipogenic effects of a high concentration of olanzapine, whereas a 10 *μ*M concentration of MK-886 potentiated the adipogenic effect of low concentrations of olanzapine.

FLAP is an important regulatory protein of the 5-LOX enzymatic pathway. In association with 5-LOX protein, FLAP regulates the synthesis of biologically active lipids, leukotrienes, and lipoxins [19, 25]. Historically, this pathway has been studied for its role in inflammation [16] and also for its putative role in central nervous system disorders such as Alzheimer's disease [26–31] and obesity [12–14, 17, 18]. It has been suggested that obesity is accompanied by adipose tissue dysfunction associated with a state of chronic mild inflammation evidenced by infiltration of inflammatory cells into adipose tissue [13, 32]. In fact, studies on human and mouse obesity have demonstrated an increased presence of FLAP in the obese adipose tissue [14, 20], which was parallel with macrophage infiltration [14]. Furthermore, treatment with a 5-LOX inhibitor was shown to be capable of reducing the obesity-associated inflammation [12].

At the cellular level, multiple mechanisms including lipoxygenases are involved in differentiation of preadipocytes into adipocytes and in their intracellular lipid accumulation [33]. Using a previously established model of olanzapine-triggered adipogenesis in 3T3-L1 cells [10], we confirmed earlier findings that 50 *μ*M olanzapine (and to a lesser extent also 0.5–5.0 *μ*M olanzapine) significantly increases cellular lipid content. It was previously shown that this effect of olanzapine can be attributed to a drug-induced expression of an SREBP-1 regulatory protein (sterol regulatory element binding protein-1) [10]. SREBP-1 was also upregulated in 3T3-L1 cells treated with another SGAD, clozapine [11]. In our experimental conditions, the adipogenic effect of olanzapine treatment was accompanied by a reduced expression of FLAP mRNA and protein, whereas the expression of 5-LOX was not significantly altered. The effect of olanzapine on FLAP content was not immediate; for example, it was not observed 24 h after the initiation of adipocyte differentiation, and it may have been set in motion by an initial lipid accumulation caused by an action of olanzapine on targets such as SREBP-1. In fact, it has been reported that a fat overload per se can suppress FLAP expression [22].

Functional implications of FLAP protein in adipocytes have been suggested by the results of *in vitro* and *in vivo* experiments with a FLAP inhibitor Bay-X-1005 [14]. This drug not only reduced the inflammatory responses in adipocytes (e.g., it caused a downregulation of inflammatory markers such as tumor necrosis factor-*α* and interleukin-6) but also *in vivo* Bay-X-1005 prevented hepatic steatosis created in a mouse model of dietary obesity [14]. In our experimental conditions, the nanomolar and low micromolar concentrations of MK-886 that are selective for FLAP inhibition reduced lipid content of cells treated with a high concentration of olanzapine. It was proposed that a FLAP-mediated synthesis of 5-LOX products in adipocytes may lead to low-grade inflammation with lipid dysfunction and adiposity [14]. In addition, it was shown that pharmacological inhibition of lipoxygenases may interfere with adipocyte differentiation and adipogenesis [3]. Our results suggest that a FLAP-dependent lipoxygenase pathway may be involved in the cellular actions of olanzapine on adipocytes.

On the other hand, we found that in contrast to FLAP-specific low concentrations of MK-886, a higher 10 *μ*M concentration of MK-886 enhanced the adipogenic effect of a low (e.g., 0.5 *μ*M) concentration of olanzapine. This action of MK-886 may be a FLAP-unrelated effect of the drug. Namely, it was shown that at 10 *μ*M MK-886 acts as a potent inhibitor of the peroxisome-proliferator-activated receptor-alpha (PPAR*α*) [34]. Hence, it is possible that PPAR*α* inhibition facilitates the adipogenic effects of olanzapine. Interestingly, we found that exposure of differentiating 3T3-L1 cells to MK-886 per se did not cause significant alterations in intracellular lipid content and that this drug altered the adipogenic effects of olanzapine in a concentration-dependent manner. Considering significant differences between the *in vitro* and *in vivo* models of drug actions on adipocytes, subsequent *in vivo* studies with olanzapine and specific FLAP inhibitors are required to evaluate the significance of our *in vitro* observations.

In conclusion, our results suggest that FLAP-mediated modifications of the 5-LOX system may be considered as a putative mechanism involved in the signaling of lipid dysfunction not only in conditions of inflammation but possibly in SGAD-related metabolic alterations and adiposity. Furthermore, the observed effects of MK-886 on cellular (e.g., adipocytes) effects of SGADS may involve both FLAP-specific and FLAP-unrelated (e.g., PPAR*α*) signaling pathways. We hypothesize that the known polymorphism in the genes of the human FLAP/5-LOX system [16] could play a role in setting a variable individual susceptibility to the peripheral/cellular metabolic side effects of SGADs. This hypothesis could be directly tested in a clinical setting.

## Figures and Tables

**Table 1:** Effect of low concentrations of MK-886 in the presence of 50 *µ*M olanzapine on intracellular lipid content.

Treatment (5 days)	Lipid content^a^
Olanzapine + Vehicle	8.3 ± 0.20
Olanzapine + 0.1 *µ*M MK-886	7.5 ± 0.15*
Olanzapine + 1.0 *µ*M MK-886	7.3 ± 0.15**
Olanzapine + 3.0 *µ*M MK-886	7.2 ± 0.08**

^a^Optical density readings at 520 nm × 10 (mean ± SEM; *n* = 3).

*P < 0.05.

**P < 0.01.

## Abbreviations

DMEM: Dulbecco's modified Eagle's medium
FBS: Fetal bovine serum
FLAP: Five lipoxygenase activating protein
IBMX: 3-Isobutyl-1-methylxanthine
5-LOX: 5-Lipoxygenase
SGADs: Second-generation antipsychotic drugs
SREBP-1: Sterol regulatory element binding protein-1.
